# Determination of patient-specific trajectory for biaxially rotational dynamic-radiation therapy using a new O-ring-shaped image guided radiotherapy system

**DOI:** 10.1016/j.phro.2025.100698

**Published:** 2025-01-20

**Authors:** Hideaki Hirashima, Hiroki Adachi, Tomohiro Ono, Mitsuhiro Nakamura, Yuka Ono, Takahiro Iwai, Michio Yoshimura, Takashi Mizowaki

**Affiliations:** a Department of Radiation Oncology and Image-Applied Therapy, Graduate School of Medicine, Kyoto University, 54 Kawahara-cho, Shogoin, Sakyo-ku, Kyoto 606-8507, Japan; b X-ray Therapy Division, Therapy System Business, Healthcare Business Group, Hitachi High-Tech Corporation, Tokyo, Japan; c Department of Radiation Oncology, Shiga General Hospital, Shiga, Japan; d Department of Advanced Medical Physics, Graduate School of Medicine, Kyoto University, 53 Kawahara-cho, Shogoin, Sakyo-ku, Kyoto 606-8507, Japan

**Keywords:** Patient-specific trajectory search, Dijkstra algorithm, Pancreatic cancer, BROAT-RT

## Abstract

**Background and purpose:**

This study developed a trajectory search method for biaxially rotational dynamic-radiation therapy (BROAD-RT) using a new O-ring-shaped linac, aimed at identifying a patient-specific trajectory in a commercial treatment planning system. Subsequently, its efficacy in the treatment of pancreatic cancer was assessed.

**Materials and methods:**

BROAD-RT is a beam delivery technique in which the gantry and O-ring are simultaneously rotated around two axes. A beam’s eye view-based structure map was generated, and the Dijkstra algorithm was then applied to explore the BROAD-RT for minimizing radiation doses to critical organs in RayStation. This procedure was evaluated in 10 patients with pancreatic cancer. For each patient, two different plans were created: volumetric modulated arc therapy (VMAT) plan with coplanar and BROAD-RT trajectory. The effects of different trajectories on the plan and dosimetric indices were assessed for each delivery technique.

**Results:**

The mean modulation complexity score for VMAT (MCS_v_) and aperture area (AA) (×10^3^ cm^2^) were 0.3 ± 0.0 and 24.8 ± 3.9 for the coplanar trajectory and 0.4 ± 0.1 and 35.2 ± 7.1 for the BROAD-RT trajectory, respectively, with both MCS_v_ (p = 5 × $10^{-5}$) and AA (p = 0.0002) values significantly higher for the BROAD-RT trajectory. Dose difference between the coplanar and BROAD-RT trajectories reduced the dose to the stomach and duodenum.

**Conclusions:**

Our study conducted an automated search for patient-specific BROAD-RT trajectory using a new O-ring-shaped linac and implemented these trajectories in RayStation. Dose distributions were reduced in the intermediate-dose regions with BROAD-RT trajectory.

## Highlights

• Biaxially Rotational Dynamic-Radiation Therapy (BROAD-RT) is achievable.
• Patient specific BROAD-RT trajectory automatically determined by Dijkstra algorithm.
• Plan and dosimetric indices compared between coplanar and BROAD-RT trajectories.
• Plan and dosimetric indices in the BROAD-RT trajectory significantly improved.

## 1 Introduction

Recent advances in non-coplanar radiotherapy emphasize its relevance in modern cancer treatment, particularly in stereotactic radiosurgery and stereotactic body radiation therapy [1]. Studies have shown that additional degrees of freedom can enhance the therapeutic ratio, either by escalating the dose to the target or reducing the dose to critical organs at risk (OARs) [2], [3], [4], [5], [6].

In recent years, dynamic non-coplanar volumetric modulated arc therapy (VMAT) has garnered increased attention for its superior dose distribution. There are two ways to achieve dynamic non-coplanar VMAT: dynamic trajectory radiation therapy (DTRT) and biaxially rotational dynamic-radiation therapy (BROAD-RT). DTRT employs non-coplanar arc beam arrangements with simultaneous gantry and couch rotation, which can be realized with C-arm linac [7], [8], [9], [10], [11], [12]. It reduces doses to the OAR by delivering radiation beams from angles that avoid direct entry through the OAR while maintaining the target dose compared to static non-coplanar beam alignment in many disease sites [7], [8], [9], [10], [11], [12]. Conversely, BROAD-RT performs VMAT by simultaneously rotating the gantry and the O-ring using an O-ring linac [13], [14], [15], [16], [17], [18]. Unlike coplanar VMAT, one of the advantages of BROAD-RT is its ability to continuously rotate the gantry and O-ring without requiring patient couch adjustments [13], [14], [15], [16], [17], [18]. These approaches have also been revealed for improving the dose distribution across various treatment sites [7], [8], [9], [10], [11], [12], [13], [14], [15], [16], [17].

Determining the patient-specific trajectory plan reduces the dose to the OAR and improves the target conformity compared with coplanar VMAT [7], [8], [9], [10], [11], [12]. Studies utilizing C-shape linacs have proposed various trajectory optimization methods, including geometrical [7], [8], [9] and fluence-based approaches [10], [11], [12], which have shown superior dose distribution compared to coplanar VMAT. However, these methods have not been implemented in clinical practices, since not being commercialize products [7], [8], [9], [10], [11], [12]. Moreover, without regulatory approval as medical devices, their clinical application remains prohibited.

This study aimed to develop patient-specific trajectory strategies for BROAD-RT in pancreatic cancer cases and evaluate OAR sparing in comparison with coplanar VMAT. Furthermore, BROAD-RT deliverability and dosimetric accuracy were evaluated utilizing a commercial O-ring linac and treatment planning system (TPS).

## 2 Methods

### 2.1 Features of the OXRAY system

**Fig. 1:**
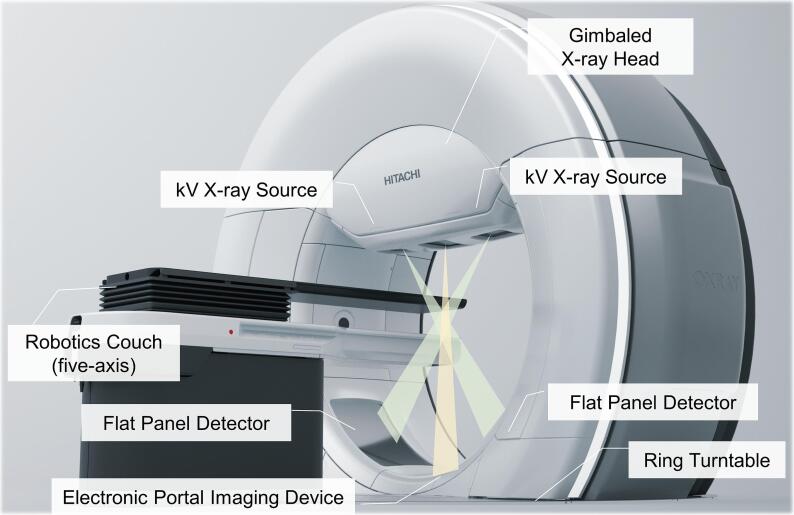
Appearance of the OXRAY system. The OXRAY system includes a gimbaled X-ray head, a dual kV X-ray imaging subsystem, an electronic portal imaging device, and a robotic treatment couch with five degrees of freedom (i.e., three translational axes and two rotational axes, pitch, and roll) for patient set-up correction.

In this study, non-coplanar rotational treatment delivery was explored using a revised version of the Vero4DRT-system (Mitsubishi Heavy Industries Ltd., Tokyo, Japan), the OXRAY system (Hitachi High-Tech Ltd., Tokyo, Japan) (Fig. 1) [19]. The comparison of machine specifications between Vero4DRT and OXRAY is shown in Supplementary Materials **and** Table S1.

### 2.2 Beams eye view-based structure map generation and trajectory exploring algorithm

**Fig. 2:**
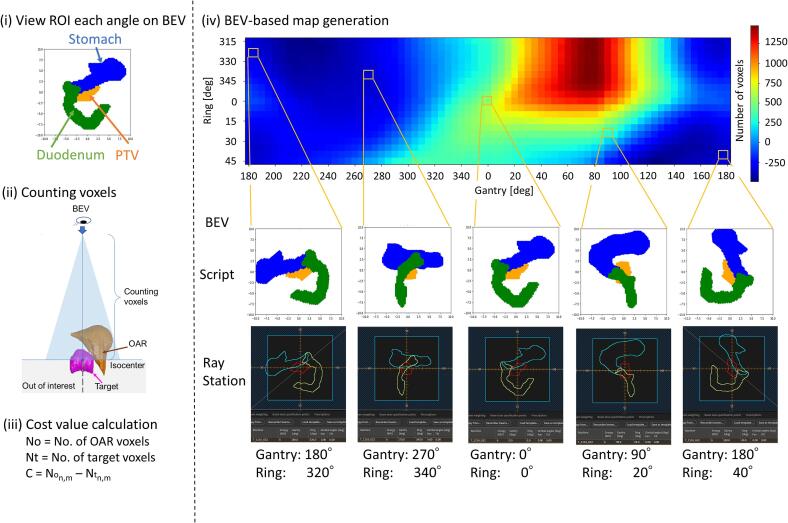
Workflow of beams eye view (BEV)-based structure map generation. The procedure is as follows: (i) view the region of interest (ROI) of each gantry and O-ring angle on BEV, (ii) count target and organ ROI voxels until isocenter plane, (iii) cost value calculation, and (iv) generate a BEV-based structure map. The upper figure on the BEV-based map shows the color map calculating the number of voxels on BEV at each gantry and O-ring position. The lower figure on the BEV-based map shows script-based BEV and BEV in RayStation. Positive values in the BEV-based structure map indicate OARs predominantly in the MLC field, whereas negative values indicate PTV predominantly in the MLC field along the PTV geometry identified in the BEV.

We devised a geometry-based approach that was created by a beam’s eye view (BEV)-based structure map using a Python script in RayStation (ver. 2023B; RaySearch Laboratories, Stockholm) to determine the patient-specific trajectory (Fig. 2). At each source position for the BEV, a Python script was employed to identify the region of interest (ROI) within the planning target volume (PTV) to determine the patient-specific trajectory for the target.

The gantry and O-ring rotation was set from −180° to 180°, while the O-ring rotation range varied from −45° to 45° considering collision, both adjusted in 5° increments to account for potential collisions. This resulted in a total of 1,296 control points (72 gantry points × 18O-ring points), which represent all possible combinations of gantry and O-ring angles. Manipulation points, defined as gantry and O-ring angle positions, where the direction of O-ring rotation can be altered, were selected from these control points. Details are described in Section 2.3. Additionally, to define collision regions between the linac and patient, we used computer-aided design to determine allowable gantry and O-ring rotation angles [17]. The resulting cost values were visualized in a structure map (Fig. 2).

For each position of the radiation source, we identified the voxels that intersected with the beam path, which is conical shaped where the base approximates the outline of the PTV as viewed from the BEV. We then calculated the number of voxels from the body surface to the isocenter, considering the PTV and OARs. The number of PTV and OAR voxels within the MLC field for the PTV geometry was counted in the BEV. Notably, voxel counts were measured up to the isocenter to provide a clear representation of the depth of voxels involved. In this study, the stomach and duodenum were specifically designated as OARs. The cost of each control point for each source position, which determined the gantry and the O-ring angle, was calculated using the following formula:

(1) $$\mathrm{Cost}={\mathrm{No}}_{n,m}-{\mathrm{Nt}}_{n,m},\;\;\;\;\;$$

where *No_n,m_* denotes the number of OAR voxels intersected by the BEV at each gantry (*n*) and O-ring angle (*m*), and *Nt_n,m_* corresponds to the total number of PTV voxels in the BEV at each gantry and O-ring control point. Regarding weight setting, we chose not to apply different weights to the target and OARs. Introducing multiple weights would increase computational complexity and compromise the model’s simplicity. We also excluded considerations such as exit dose and OAR thickness to maintain simplicity. The primary objective of this study was to develop a practical and robust optimization method. Therefore, we focused on the entrance dose volume at each angle, aiming to balance adequate target coverage with OAR avoidance, while keeping the cost function straightforward.

### 2.3 Selection of patient-specific trajectory

**Fig. 3:**
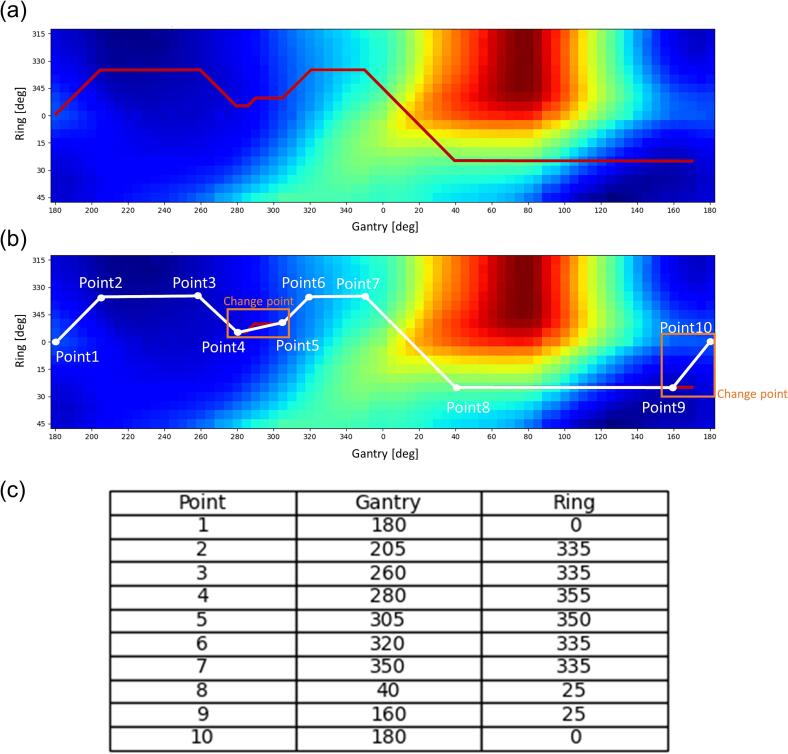

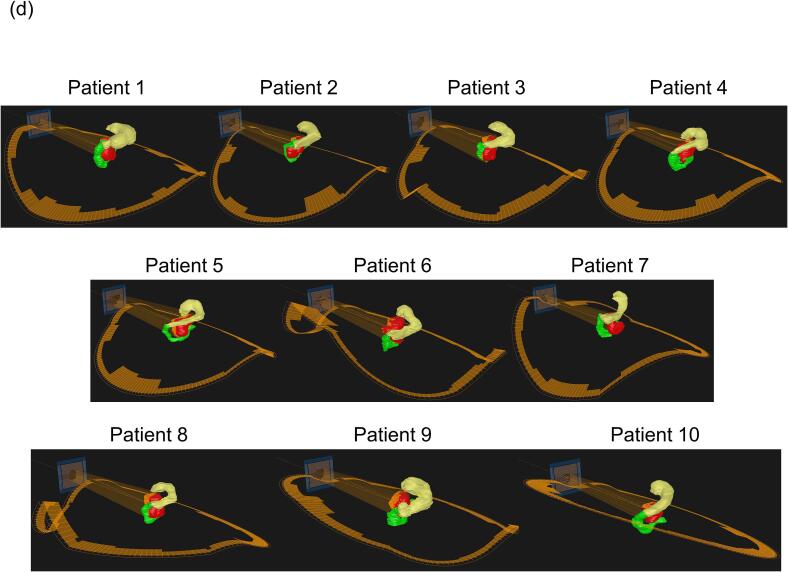
Patient-specific trajectory with Dijkstra algorithm on BEV-based structure map. (a) Path trajectory on the structure map, (b) deliverable patient-specific trajectory plot, and (c) deliverable manipulation points, (d) BORAD-RT trajectories in each patient. Plotted points are summarized in a table. In the clinical workflow, plotted points in the table are entered while editing for treatment planning system trajectory.

Subsequently, trajectory optimizations were conducted using the Dijkstra algorithm [20], which determined the trajectory with minimum cost based on the structure map. This algorithm was developed considering the traveling salesman problem, which is a classic optimization problem where the goal is to determine the shortest possible route that visits a set of points once and returns to the origin, wherein the gantry/O-ring was represented as several control points connected to each other. The shortest paths from a starting control point to all other control points were calculated in a weighted graph. Fig. 3**a** shows the least-cost trajectory generated using the Dijkstra’s algorithm.

This study aimed to enhance beam delivery efficiency by minimizing unnecessary interruptions caused by continuous adjustments of the O-ring during beam delivery. The trajectory’s direction was defined by the Supplementary Material.

### 2.4 Patient selection, contouring and treatment planning

We retrospectively analyzed 10 patients with borderline resectable pancreatic cancer who were treated using a single full-arc VMAT with breath-hold technique between January 2020 and December 2022. This study was approved by our Institutional Review Board (R1446-2) and adhered to the ethical principles outlined in the Helsinki Declaration. Targets and OARs were delineated by certified radiation oncologists, based on a previously reported institutional protocol [21], [22].

In this context, Dijkstra’s trajectory refers to the BROAD-RT trajectory. For each patient, two different single full-arc plans were created using RayStation (ver. 2023B): VMAT plan with coplanar and BROAD-RT trajectory. VMAT plans were created for both the coplanar and BROAD-RT trajectory RayStation (ver. 2023B). The treatment plan was designed to meet the clinical goals and dose-volume constraints specified in Supplementary Table S2.

### 2.5 Plan evaluation

The trajectory score (TS) was quantitatively assessed for each trajectory, as follows:

(2) $$TS=\;\frac{1}{n}\sum_{i=1}^{n}\sum_{j=1}^{m}\frac{Cost\left(i,j\right)}{Max\;Cost\left(i\right)},$$

where *n* and *m* represent the total number of gantry and O-ring control points, and *i* and *j* denote the values of the gantry and O-ring control points on the structure map. Further, *Cost*(*i, j*) represents the structure map value at (*i, j*), and Max *Cost*(*i*) denotes the maximum map cost. TS was scored on a scale of 0–1 for each gantry angle, enabling independent evaluation irrespective of the ROI volume. Path length was assessed by evaluating the Euclidean distance of each trajectory, with higher values indicating longer paths and lower values indicating shorter paths.

The plan and dosimetric indices were evaluated for both the coplanar and BROAD-RT trajectory plans. The theoretical delivery time for the VMAT plans was calculated by the TPS for both the coplanar and BROAD-RT trajectories. Plan parameters such as monitor unit (MU), Paddicks conformity index (CI) [23], modulation complexity score for VMAT (MCS_v_) [24], and aperture area (AA) were assessed. The VMAT plans with the coplanar and BROAD-RT trajectories were evaluated for dosimetric parameters. The dose covering 98% (D_98%_) of the PTV and the maximum dose (D_max_) were considered for the target. Volumes receiving 42 Gy (V_42 Gy_), 39 Gy (V_39 Gy_), 36 Gy (V_36 Gy_), 30 Gy (V_30 Gy_), 20 Gy (V_20 Gy_), and 10 Gy (V_10 Gy_) were evaluated for the stomach and duodenum. Dosimetric indices of D_2 cc_, V_20 Gy_, and mean dose were assessed for the PRV of the spinal cord (PRV_Spinal Cord), kidney, and liver. The Paddicks CI and ratio of the X% isodose line volume to the PTV volume (RX%) were evaluated. Statistical analyses were conducted using a paired *t*-test, with statistical significance set at p < 0.05.

## 3 Results

The mean ± standard deviation (SD) of TS in trajectory indices were 0.69 ± 0.04 for the coplanar trajectory and 0.36 ± 0.04 for the BROAD-RT trajectory, with a significant reduction in TS observed for the BROAD-RT trajectory (p = 1 × $10^{-8}$) (Table 1). The mean ± SD of the Euclidean distance in the trajectory indices were 1.0 ± 0.0 for the coplanar trajectory and 1.2 ± 0.1 for the BROAD-RT trajectory, with a significant long in the Euclidean distance observed for the BROAD-RT trajectory (p = 2 × $10^{-6}$) (Table 1). The mean ± SD of delivery time for the coplanar and BROAD-RT trajectories were 69.8 ± 11.1 s for the coplanar trajectory and 94.1 ± 8.0 s for the BROAD-RT trajectory, with the BROAD-RT trajectory requiring significantly more time (p = 0.0004). The mean ± SD of MU were 1083 ± 141 MU for the coplanar trajectory and 799 ± 95 MU for the BROAD-RT trajectory, with the BROAD-RT trajectory showing a significantly lower MU (p = 3 × $10^{-5}$). The mean MCS_v_ and AA (×10^3^ cm^2^) were 0.3 ± 0.0 and 24.8 ± 3.9 for the coplanar trajectory and 0.4 ± 0.1 and 35.2 ± 7.1 for the BROAD-RT trajectory, respectively, with both MCSv (p = 5 × $10^{-5}$) and AA (p = 0.0002) values significantly higher than the BROAD-RT trajectory. The computation time required for trajectory determination was less than 1 min, and the planning time needed for each method to meet dose constraints was less than 30 min. Fig. 3**d** shows the BROAD-RT trajectory determined by the Dijkstra algorithm.

**Table 1:** Mean ± standard deviation of trajectory score, Euclidean distance, delivery time, plan quality, and dosimetric indices in VMAT plans with coplanar and BROAD-RT trajectories.

		coplanar	Dijkstra	p value
Manipulation point [median (min − max)]			8 (5–10)	
Trajectory score		0.69 ± 0.04	0.36 ± 0.04	1×$10^{-8}$
Euclidean distance		1.0 ± 0.0	1.2 ± 0.1	2×$10^{-6}$
Delivered time [s]		69.8 ± 11.1	94.1 ± 8.0	0.0004
**Plan index**				
MU		1082.5 ± 141.0	798.7 ± 94.7	3×$10^{-5}$
MCSv		0.3 ± 0.0	0.4 ± 0.1	5×$10^{-5}$
AA	(×10^3^ cm^2^)	24.8 ± 3.9	35.2 ± 7.1	0.0002
**Dosimetric index**				
PTV	D_98%_ [Gy]	36.3 ± 0.3	36.3 ± 0.3	1.00
	D_max_ [Gy]	45.9 ± 0.2	45.8 ± 0.2	1.00
Stomach	V_42 Gy_ [cm^3^]	0.0 ± 0.0	0.0 ± 0.0	1.00
	V_39 Gy_ [cm^3^]	0.1 ± 0.1	0.1 ± 0.2	0.69
	V_36 Gy_ [cm^3^]	1.6 ± 1.8	1.6 ± 1.8	0.87
	V_30 Gy_ [cm^3^]	5.8 ± 4.7	5.2 ± 4.6	0.63
	V_20 Gy_ [cm^3^]	23.8 ± 14.0	21.9 ± 13.1	0.92
	V_10 Gy_ [cm^3^]	69.5 ± 29.7	55.9 ± 26.6	0.19
Duodenum	V_42 Gy_ [cm^3^]	0.0 ± 0.0	0.0 ± 0.0	0.34
	V_39 Gy_ [cm^3^]	0.2 ± 0.3	0.1 ± 0.2	0.49
	V_36 Gy_ [cm^3^]	9.3 ± 4.7	8.2 ± 5.1	0.35
	V_30 Gy_ [cm^3^]	17.3 ± 6.8	15.9 ± 7.3	0.49
	V_20 Gy_ [cm^3^]	29.3 ± 9.2	28.5 ± 11.6	0.30
	V_10 Gy_ [cm^3^]	46.4 ± 16.3	44.7 ± 14.8	0.33
PRV_Spinal Cord	D_2 cc_ [Gy]	24.6 ± 2.8	23.3 ± 3.0	0.32
Right kidney	V_20 Gy_ [cm^3^]	0.01 ± 0.01	0.01 ± 0.01	0.22
	Mean dose [Gy]	6.5 ± 1.2	6.4 ± 1.5	0.82
Left kidney	V_20 Gy_ [cm^3^]	0.04 ± 0.04	0.04 ± 0.03	0.63
	Mean dose [Gy]	7.7 ± 8.7	8.2 ± 7.8	0.03
Liver	Mean dose [Gy]	5.3 ± 2.8	6.3 ± 3.8	0.07
**Conformity index**		0.83 ± 0.05	0.84 ± 0.03	1.00
R70%		2.61 ± 0.28	2.58 ± 0.24	1.00
R50%		4.94 ± 0.53	4.87 ± 0.41	1.00
R30%		12.65 ± 1.67	12.17 ± 1.83	1.00

Abbreviations: BROAD-RT, Biaxially ROtAtional Dynamic-Radiation Therapy; VMAT, volumetric modulated arc therapy; PTV, planning target volume; PRV, planning organ-at-risk volume; MU, monitor unit; MCSv, modulation complexity score for VMAT; AA, aperture area; RX%, ratio of X% isodose line volume to PTV volume.1.

**Fig. 4:**
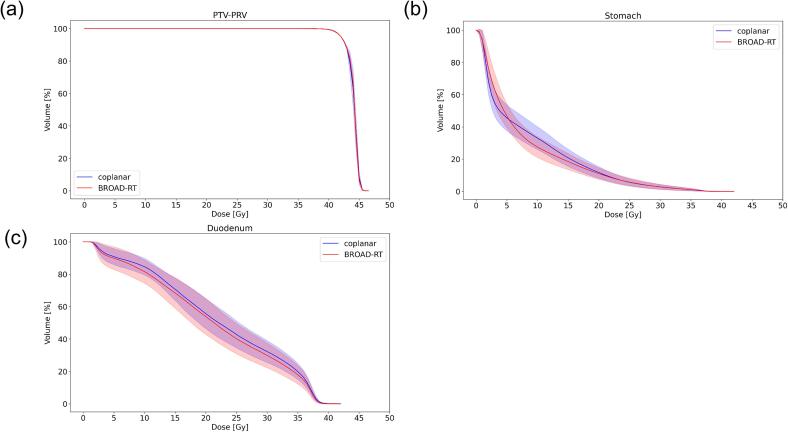
Mean DVH and 95% confidence level of VMAT plan between coplanar and BROAD-RT trajectories for (a) DVH of PTV-PRV, (b) stomach, and (c) duodenum. Blue and red lines indicate the mean DVH of the coplanar and BROAD-RT trajectories. (For interpretation of the references to color in this figure legend, the reader is referred to the web version of this article.)

**Fig. 5:**
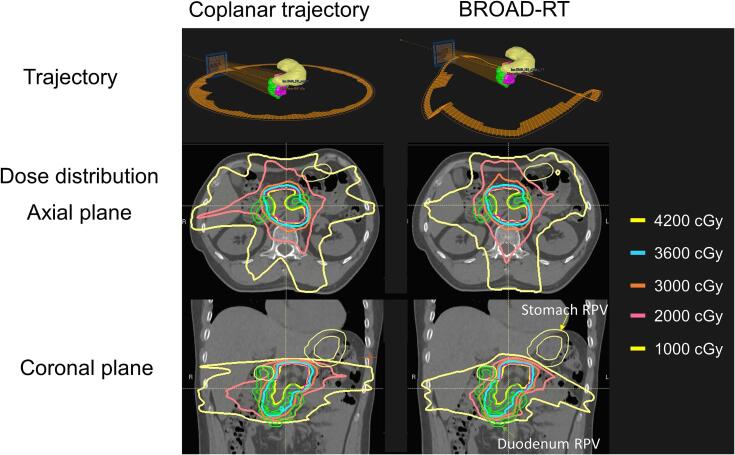
Dose distribution in representative patient between coplanar and BROAD-RT trajectories. Figure visualized dose distribution (coplanar and BROAD-RT trajectories) and OARs (stomach and duodenum).

Dosimetric indices were satisfied in all trajectory plans for the VMAT plan. The VMAT plan with the coplanar trajectory exhibited a statistically significantly higher mean dose to the left kidney compared to BROAD-RT (p = 0.03). Fig. 4 illustrates the average dose-volume histogram and the 95% confidence level between the two trajectories. Fig. 5 displays the representative dose distributions for the two trajectories. The intermediate-dose region for the stomach and duodenum decreased compared to the coplanar trajectory while maintaining the target dose. In the stomach, the V_10Gy_ in the BROAD-RT’s plan was reduced by 10% compared to that in the coplanar trajectory. The BROAD-RT trajectory resulted in lower doses to the PRV than the coplanar trajectory.

Conformity indices, such as Paddicks CI, R30%, R50%, and R70%, for the two trajectories did not vary significantly (Table 1). The dose-volume histogram comparison indicates that the BROAD-RT trajectory plan statistical significantly reduced the dose to selected OARs while maintaining target coverage comparable to the coplanar trajectory plan (Fig. 4).

## 4 Discussion

This study demonstrates the application of the Dijkstra algorithm to determine the patient-specific trajectory for BROAD-RT using a new O-ring-shaped IGRT system linac. The proposed technique is particularly beneficial in scenarios requiring high-dose delivery to the target while minimizing exposure to OARs, such as in hypofractionated radiotherapy applications. Our method employs an automated approach for selecting non-coplanar trajectories using a trajectory optimization algorithm to compare dose distributions for pancreatic cancer in a clinical context. This approach is unlike earlier research that relied on manual static beam orientation selection and emphasizes the unique focus of our work on clinical implementation. By introducing a cost function based on structure map, in our experiments, we simplified and streamlined the process, optimizing beam direction to minimize doses to OARs. The process begins with the creation of a BEV-based structure map for each patient. We then applied the Dijkstra algorithm to identify the trajectory with the lowest cost through this map. In the current approach, the isocenters are positioned at the center of the PTV. This approach is based on the rationale that isocenters are conventionally placed at the center of the target during treatment planning. Deviation from this positioning could affect the consistency of the map score. However, this study did not address cases where the isocenter is located outside the PTV center, as such occurrences were infrequent and considered negligible. Selecting a non-coplanar arc trajectory allowed for more flexible beam orientation compared to coplanar arcs, potentially reducing OAR doses and mitigating treatment plan complexity.

The treatment plan utilizing the BROAD-RT trajectories demonstrated improvement in plan indices, including MU and MCS_v_, compared to the coplanar trajectory. This reflects the complexity of treatment planning and highlights the potential for streamlining the process through individually tailored trajectories. By contrast, coplanar trajectories often require intricate optimization due to their consistent paths, regardless of the patient’s organ position and shape. From these metrics, the robustness of the plan is considered to have shown improvement. Effective beam orientation optimization not only simplifies treatment planning but also ensures unobstructed beam delivery [1], [14], [25]. This study optimized the beam incidence direction by accounting for the unique anatomical features and positions of each patient’s organs, leading to a patient-specific treatment plan.

The path length determined by the BROAD-RT trajectory was longer than that of the coplanar trajectory because of non-coplanar trajectory, resulting in a longer delivery time. In terms of delivery time, however, previous studies reported treatment times ranging from 3 to 8 min for doses of 2 to 12 Gy per fraction in DTRT [7], [8], [9], [10], [11], [12]. This is much longer than the approximately 1.5 min (2.8 Gy/fraction) for BROAD-RT observed in our study. Additionally, no constraints, such as being limited to the research mode, exist. Therefore, the extended delivery time associated with the BROAD-RT trajectory does not pose a barrier to its clinical applications.

The BROAD-RT trajectory reduces the intermediate-dose regions for the stomach, duodenum, and kidney, such as V_20 Gy_, V_10 Gy_, and the mean dose, compared with the coplanar arc trajectory (Table 1). Previous research correlated low- and intermediate-dose regions of the stomach and duodenum with gastrointestinal toxicity [26]. Therefore, a simple reduction of these dose ranges to OARs (stomach and duodenum) can be clinically advantageous. In our case, no significant differences were observed in OARs of the VMAT plan between the coplanar and BROAD-RT trajectories due to intensity modulation. Nevertheless, the intermediate-dose region in the stomach and duodenum was effectively reduced while maintaining the target dose. This is in contrast to the coplanar trajectory, due to the selection of a trajectory that avoided these organs. The optimized trajectories did not explicitly optimize intensity modulation but were determined based on an optimization method that prioritizes OAR avoidance as a criterion for selecting rotational trajectories. Tailoring the selection of OARs based on individual cases may potentially reduce the irradiation dose to the OARs. The conformity indices of Paddicks CI, R70%, R50%, and R30% for the BROAD-RT trajectory were not significantly different compared to the coplanar trajectory. However, they showed a trend toward higher dose conformity (Fig. 5 and Table 1). Several studies have demonstrated that non-coplanar delivery methods, such as 4π therapy, static non-coplanar beams, and DTRT, improve both target dose distribution and conformity in OAR doses [1], [2], [3], [4], [5], [6], [7], [8], [9], [10], [11], [12], [13], [14], [15], [16], [17]. As a result, the dose distribution maintains a more uniform and predictable pattern, reducing irregularities and reducing the plan complexity.

Burghelea et al. reported that there was no clear superiority between Dynamic WaveArc (DWA) and coplanar VMAT in dose distribution comparisons for pancreatic cancer [15]. However, considering that Vero4DRT cannot perform a collimator rotation, DWA was recommended in practice for targets with irregular shapes [15]. Additionally, they indicated that the trajectories used were pre-defined in the TPS, suggesting that patient-specific trajectory selection could further improve the dose distribution. Compared to Vero4DRT, OXRAY enables improved O-ring rotation speed and intensity modulation, making it possible to create treatment plans using patient-specific trajectories. Consequently, in this study, unlike the results in their paper, it became clear that the dose distribution of BROAD-RT improved compared to coplanar VMAT. This personalized treatment planning approach accounts for individual anatomical differences and variations in the location of OARs, thereby aiming to improve treatment outcome.

Our research demonstrates that patient-specific non-coplanar trajectory plans reduce the dose to OAR and improve the target conformity compared to the coplanar trajectory plans, consistent with several previous reports [7], [8], [9], [10], [11], [12]. Therefore, developing these techniques should consider their clinical practicality. Although studies on determining patient-specific non-coplanar trajectories using a C-shaped linac in research mode and a research version of TPS are ongoing, their feasibility in clinical practice has not been addressed to date [7], [8], [9], [10], [11], [12]. Hence, the strength of our approach lies in its seamless compatibility with a commercial TPS and its feasibility for clinical use, in stark contrast to DTRT [7], [8], [9], [10], [11], [12]. Therefore, our method can be considered a viable approach for implementing patient-specific trajectories in clinical practice. For clinical implementation, we envision a workflow where structure maps are created using scripts prior to treatment planning, and trajectory selection would be based on these maps. Regarding potential limitations, one key consideration is the verification process to ensure that the generated trajectories can be properly read and executed by the treatment machine’s record and verify system. Looking toward future developments, our approach using scripts enables various improvements and modifications to both structure mapping and trajectory optimization methods for clinical implementation. What makes this particularly interesting is that this advanced technical development is currently uniquely achievable through the combination of RayStation and OXRAY systems. This exclusivity provides an opportunity for pioneering developments in the field.

This study has three limitations: First, the study was limited by its sample size and disease site. Although this investigation focused exclusively on pancreatic cancer, the proposed method is applicable to all disease sites. Further research with a larger patient cohort is required to confirm the effectiveness of the proposed method. Second, the number of selected organs and irradiation arcs was limited during the generation of the structure map and set to one arc during treatment planning. In our institution, a single full-arc VMAT technique with breath-hold was adopted for managing tumor respiratory motion in pancreatic cancer. Consequently, this study conducted a comparative analysis of dose distribution using single-arc VMAT technique. In our method, multiple organs can be selected, and trajectory selection avoids the selected organs. However, it remains unknown whether a trajectory with multiple-organ selection can consistently reduce the doses to the selected organs. Consequently, in our experiments, the number of organs was limited for a simplified evaluation. Further, although multiple arcs were commonly employed in the treatment planning to improve the dose distribution, the evaluation method excluded the effect of the number of arcs to assess the usefulness of trajectory optimization based on a structure map. Third, we examined only one method for trajectory optimization, so we cannot determine if this is optimal. However, we clarified that we have demonstrated the superiority of the dose distribution using our proposed trajectory method compared to coplanar trajectories. Fourth, we exclusively assessed OXRAY. Even though the proposed method can be applied to all disease sites and other linacs, it does not encompass or evaluate the dosimetric effects on BROAD-RT. Further studies should investigate the effectiveness of our methodology across different disease sites and linacs, with additional optimization and validation needed to ensure its safe and effective implementation in clinical practice.

In conclusion, our study aimed to automatically search for patient-specific non-coplanar trajectories using a new O-ring-shaped IGRT system. This method was successfully implemented in a commercial TPS. By targeting specific areas while preserving intermediate-dose regions in critical organs, our approach reduced the dose distribution in these regions, consequently improving the overall quality of the treatment plan. Further research is required to validate the effectiveness and safety of this method in a larger cohort of patients.

## CRediT authorship contribution statement

**Hideaki Hirashima:** Conceptualization, Data curation, Formal analysis, Investigation, Methodology, Software, Writing – original draft. **Hiroki Adachi:** Software, Formal analysis, Investigation, Writing – review & editing. **Tomohiro Ono:** Conceptualization, Data curation, Formal analysis, Resources, Writing – review & editing. **Mitsuhiro Nakamura:** Conceptualization, Funding acquisition, Project administration, Supervision, Visualization, Writing – review & editing. **Yuka Ono:** Resources, Writing – review & editing. **Takahiro Iwai:** Writing – review & editing. **Michio Yoshimura:** Writing – review & editing. **Takashi Mizowaki:** Conceptualization, Funding acquisition, Project administration, Supervision, Visualization, Writing – review & editing.

## Declaration of competing interest

The authors declare the following financial interests/personal relationships which may be considered as potential competing interests: This study was funded by Hitachi Ltd. Takashi Mizowaki has received research grants and a scholarship donation from Hitachi Ltd. Mitsuhiro Nakamura has received a scholarship donation from Hitachi Ltd. The other co-authors are collaborative research with Hitachi Ltd.

## References

[1] Smyth G., Evans P.M., Bamber J.C., Bedford J.L. (2019). Recent developments in non-coplanar radiotherapy. Br J Radiol.

[2] Dong P., Lee P., Ruan D., Long T., Romeijn E., Yang Y. (2013). 4π non-coplanar liver SBRT: A novel delivery technique. Int J Radiat Oncol Biol Phys.

[3] Dong P., Lee P., Ruan D., Long T., Romeijn E., Low D.A. (2013). 4π noncoplanar stereotactic body radiation therapy for centrally located or larger lung tumors. Int J Radiat Oncol Biol Phys.

[4] Audet C., Poffenbarger B.A., Chang P., Jackson P.S., Lundahl R.E., Ryu S.I. (2011). Evaluation of volumetric modulated arc therapy for cranial radiosurgery using multiple noncoplanar arcs. Med Phys.

[5] Breedveld S., Storchi P.R., Voet P.W., Heijmen B.J. (2012). iCycle: Integrated, multicriterial beam angle, and profile optimization for generation of coplanar and noncoplanar IMRT plans. Med Phys.

[6] Woods K., Nguyen D., Tran A., Yu V.Y., Cao M., Niu T. (2016). Viability of non-coplanar VMAT for Liver SBRT as compared to coplanar VMAT and beam orientation optimized 4π IMRT. Adv Radiat Oncol.

[7] Fix M.K., Frei D., Volken W., Terribilini D., Mueller S., Elicin O. (2018). Part 1: Optimization and evaluation of dynamic trajectory radiotherapy. Med Phys.

[8] Bertholet J., Mackeprang P.H., Mueller S., Guyer G., Loebner H.A., Wyss Y. (2022). Organ-at-risk sparing with dynamic trajectory radiotherapy for head and neck cancer: Comparison with volumetric arc therapy on a publicly available library of cases. Radiat Oncol.

[9] Bertholet J., Mackeprang P.-H., Loebner H.A., Mueller S., Guyer G., Frei D. (2024). Organs-at-risk dose and normal tissue complication probability with dynamic trajectory radiotherapy (DTRT) for head and neck cancer. Radiother Oncol.

[10] Lyu Q., Yu V.Y., Ruan D., Neph R., O’Connor D., Sheng K. (2018). A novel optimization framework for VMAT with dynamic gantry couch rotation. Phys Med Biol.

[11] Mullins J., Renaud M.A., Serban M., Seuntjens J. (2020). Simultaneous trajectory generation and volumetric modulated arc therapy optimization. Med Phys.

[12] Wang G., Wang H., Zhuang H., Yang R. (2021). An investigation of non-coplanar volumetric modulated radiation therapy for locally advanced unresectable pancreatic cancer using a trajectory optimization method. Front Oncol.

[13] Mizowaki T., Takayama K., Nagano K., Miyabe Y., Matsuo Y., Kaneko S. (2013). Feasibility evaluation of a new irradiation technique: three-dimensional unicursal irradiation with the Vero4DRT (MHI-TM2000). J Radiat Res.

[14] Burghelea M., Verellen D., Dhont J., Hung C., Gevaert T., Van den Begin R. (2017). Treating patients with Dynamic Wave Arc: First clinical experience. Radiother Oncol.

[15] Burghelea M., Verellen D., Poels K., Hung C., Nakamura M., Dhont J. (2016). Initial characterization, dosimetric benchmark and performance validation of Dynamic Wave Arc. Radiat Oncol.

[16] Uto M., Mizowaki T., Ogura K., Miyabe Y., Nakamura M., Mukumoto N. (2017). Volumetric modulated Dynamic WaveArc therapy reduces the dose to the hippocampus in patients with pituitary adenomas and craniopharyngiomas. Pract Radiat Oncol.

[17] Ono Y., Yoshimura M., Hirata K., Ono T., Hirashima H., Mukumoto N. (2018). Dosimetric advantages afforded by a new irradiation technique, Dynamic WaveArc, used for accelerated partial breast irradiation. Phys Med.

[18] Hiraoka M., Mizowaki T., Matsuo Y., Nakamura M., Verellen D. (2020). The gimbaled-head radiotherapy system: Rise and downfall of a dedicated system for dynamic tumor tracking with real-time monitoring and dynamic WaveArc. Radiother Oncol.

[19] Stronger and more flexible X-ray radiotherapy. Hitachi Ltd. Nature Portfolio. 2024 Mar 20. https://www.nature.com/articles/d42473-023-00445-6.

[20] Dijkstra E.W. (1959). A note on two problems in connexion with graphs. Numer Math.

[21] Goto Y., Nakamura A., Ashida R., Sakanaka K., Itasaka S., Shibuya K. (2018). Clinical evaluation of intensity-modulated radiotherapy for locally advanced pancreatic cancer. Radiat Oncol.

[22] Masui T., Nagai K., Anazawa T., Sato A., Uchida Y., Nakano K. (2022). Impact of neoadjuvant intensity-modulated radiation therapy on borderline resectable pancreatic cancer with arterial abutment; a prospective, open-label, phase II study in a single institution. BMC Cancer.

[23] Paddick I. (2000). A simple scoring ratio to index the conformity of radiosurgical treatment plans. Technical note J Neurosurg.

[24] Masi L., Doro R., Favuzza V., Cipressi S., Livi L. (2013). Impact of plan parameters on the dosimetric accuracy of volumetric modulated arc therapy. Med Phys.

[25] Wild E., Bangert M., Nill S., Oelfke U. (2015). Noncoplanar VMAT for nasopharyngeal tumors: Plan quality versus treatment time. Med Phys.

[26] Nakamura A., Shibuya K., Matsuo Y., Nakamura M., Shiinoki T., Mizowaki T. (2012). Analysis of dosimetric parameters associated with acute gastrointestinal toxicity and upper gastrointestinal bleeding in locally advanced pancreatic cancer patients treated with gemcitabine-based concurrent chemoradiotherapy. Int J Radiat Oncol Biol Phys.

