# Supplementary material for: Determination of patient-specific trajectory for biaxially rotational dynamic-radiation therapy using a new O-ring-shaped image guided radiotherapy system
Source: Phys Imaging Radiat Oncol. 2025 Jan 20;33:100698. doi: 10.1016/j.phro.2025.100698 (PMC11926428; doi:10.1016/j.phro.2025.100698)
Supplement: Supplementary Data 1 [file mmc1.pdf]

Supplementary Materials

Features of the OXRAY system

The OXRAY consisted of a robust O-ring structure housing a gimbal-driven irradiation head that accommodates a compact C-band linac and a pair of orthogonal kV X-ray imaging systems. It was characterized using a pair of orthogonal kV X-ray imaging systems. The heights from the floor to the top of the O-ring, width of O-ring, and inner diameter were 299, 391, and 200 cm, respectively. The source-axis distance was 100 cm. The maximum static field size was 20 × 20 cm. Beam energies of 6 MV and 6 MV FFF were used. The treatment aperture consisted of a multi-leaf collimator (MLC) with the central 16 leaves measuring 2.5 mm in width and the outer 32 leaves measuring 5 mm in width at the isocenter. The gantry rotated within a range of ±185° at a maximum speed of 7°/s. The O-ring could rotate around its vertical axis within a range of ±60° at a maximum speed of 6°/s, doubling the speed of the Vero4DRT. As with theVero4DRT system the OXRAY system was also able to move both the rotation axes (gantry and O-ring) simultaneously. The theoretical concept of BROAD-RT was presented by Mizowaki et al. [1] under the term “3D unicursal irradiation”. BROAD-RT is commercially available under the brand names of Dynamic WaveArc® (DWA in Vero4DRT)

[2-6] and Dynamic SwingArc® (DSA in OXRAY) [7]. The comparison of machine specifications between Vero4DRT and OXRAY is shown in **Supplementary Table S1**.

Supplementary Table S1. A brief comparison of specifications between Vero4DRT and OXRAY.

	**Vero4DRT**	**OXRAY**
**Mechanical characteristics**		
Gantry rotational speed	7°/s	7°/s
O-ring rotational speed	3°/s	6°/s
Gantry stroke	±185°	±185°
O-ring stroke	±60°	±60°
**Irradiation system**		

Beam energy (dose rate)	6 MV (500 MU/min)	6 MV (600 MU/min)<br>6 MVFFF (1200 MU/min)
Field size	15 × 15 cm	20 × 20 cm
MLC width	5 mm (30 pairs)	2.5 mm (16 pairs: ±40 mm of the isocenter)<br>5 mm (32 pairs: other)
MLC speed@iso	50 mm/s	65 mm/s

Selection of patient-specific trajectory

This study aimed to enhance beam delivery efficiency by minimizing unnecessary interruptions caused by continuous adjustments of the O-ring during beam delivery. The trajectory's direction was defined by the following constraints:

(1) The O-ring angle was manually set to 0° at both the start and end points to optimize patient throughput in clinical practice. To increase the irradiation efficiency, the start and end points were set at a gantry angle ring angle of 0°. Due to the inherent properties of Dijkstra's algorithm, the starting point could be determined. However, the endpoint could not be algorithmically established. Consequently, the endpoint was manually taking the O-ring angle into consideration. The variation in trajectory score due to manual adjustment of the start and end points was within 0.05. In this study, we compared the results with coplanar trajectories, which were significantly lower using the Dijkstra algorithm, even when the start and end points were not adjusted. Therefore, the start and end points were adjusted manually from the point of view of irradiation efficiency, as there was no significant variation in the score;

(2) As the gantry rotates clockwise from -180° to +180°, the trajectory can move in either a straight or diagonal direction. Diagonal paths were restricted to a maximum of one angle square per angle in the gantry direction, in accordance with the movement rules during BROAD-RT;

(3) The number of manipulation points was limited to 10 to ensure deliverability. The rationale for limiting manipulation points to a maximum of 10 was to ensure that the O-ring could safely adjust its direction within a single rotation. This constraint was established to maintain balance between precise beam delivery and operational safety;

(4) Manual adjustments were made to address delivery constraints due to physical limitations of the machine (**Figs. 3b** and **3c**), specifically the speed limits of the gantry rotation, O-ring rotation, MLC speed, and dose rate [2].

Patient selection, contouring and treatment planning

For each patient, two different single full-arc plans were created using RayStation (ver. 2023B): VMAT plan with coplanar and BROAD-RT trajectory. VMAT plans were created for both the coplanar and BROAD-RT trajectory RayStation (ver. 2023B). Partial arc rotation was not considered in this study. This exclusion was based on the need to maintain consistency in experimental conditions; thus, a single full-arc rotation was assumed and used to compare coplanar trajectory with BROAD-RT.

A prescribed dose of 42 Gy in 15 fractions was specified to the PTV-PRV with $D_{95\%}$. Isocenter was set at the PTV center. Both the coplanar and BROAD-RT trajectory plans were generated using a BEV-based structure map. Optimization of dose distribution was conducted by RayOptimizer in RayStation (ver. 2023B). The collapsed cone algorithm (ver. 5.8) was employed as the dose-calculation algorithm. The clinical goals and dose-volume constraints are listed in **Supplementary Table S2**. The number of optimization objects and iterations for both trajectory plans were adjusted individually to satisfy the dose constraints. The dose-volume constraints for the VMAT plan were based on a previously reported institutional protocol [8].

Supplementary Table S2. Dose constraint for pancreatic cancer.

Structure	Constraint	
PTV-PRV	$D_{95\%}$	≥42 Gy
PTV	$D_{98\%}$	>36 Gy
Body	D_{max}	<48.3 Gy
Stomach/Duodenum/Bowel Large/Bowel Small	$V_{42\ Gy}$	<0.5 cm^3
	$V_{39\ Gy}$	<1 cm^3
	$V_{36\ Gy}$	<20 cm^3
PRV_Stomach/PRV_Duodenum	$V_{39\ Gy}$	<15 cm^3
	$V_{36\ Gy}$	<45 cm^3
Spinal Cord	D_{max}	<36 Gy
PRV_Spinal Cord	$D_{2\ cc}$	<39 Gy
Kidney (at least one)	$V_{20\ Gy}$	<30%
Liver	D_{mean}	<30 Gy

Abbreviations: PTV, planning target volume; PRV, planning organs at risk volume.

Quality assurance

The calculated and measured dose distributions of the VMAT plan with the coplanar and BROAD-RT trajectory were assessed according to global gamma analysis using ArcCHECK (Sun Nuclear, Melbourne, FL, USA). The criteria of gamma passing rate (GPR) were 3% of the planned maximum dose as the dose-difference criterion and 2 mm as the distance-to-agreement criterion (γ3%/2mm), with a 10% threshold; the criteria for pass and fail were ≥95% at γ3%/2mm based on AAPM-TG218 [9].

The mean ± SD of the GPR (γ3%/2 mm) was 97.1 ± 1.6% for the coplanar trajectory and 96.0 ± 1.6% for BROAD-RT, respectively. The overall RATING score was 96% (**Supplementary Table S3**) [10].

Supplementary Table S3. RATING score sheet

RATING score sheet

		Points	Applicable/ relevant	Answer yes
**Questions for the Introduction**				
	The study aim formulated by research questions			
1	Does the study have a concise and precise study aim, defined with a restricted number of interconnected questions?	10		☑
	The motivation for the research questions			
2	Has relevant up to date literature been included to support the need for the current study?	5		☑
3	Does the study address an existing knowledge gap?	10		☑
**Questions for Materials and Methods**				
4	Is the global study design adequate for answering the posed research questions?	10		☑
5	Is the global study design described in sufficient detail for others to interpret and reproduce the results?	5		☑
	Patient cohort			
6	Are the inclusion and exclusion criteria of the patient cohort described?	1	☑	☐
7	Is the clinical patient information of the cohort presented, including disease type, site(s) and clinical staging?	1	☑	☑
8	Is the included number of patients stated, explained and justified?	1	☑	☑
9	Has there been consideration of the need for ethical and/or legal approval for the study and if needed, is there a statement about this?	5		☑
	Imaging procedures			
10	Have the scanning parameters been reported in sufficient detail (image modalities, equipment model, slice thickness, voxel size, patient position (e.g. head first, supine, etc.) etc.)?	1	☑	☑
11	Has the applied immobilisation equipment been described, (e.g. vendor and type, standard settings, etc.) where relevant?	1	☑	☑
	Treatment machine and settings			
12	Have the treatment machine and relevant parameters been described with sufficient detail (model, beam energy, MLC, etc.)?	1	☑	☑
13	Have the monitor unit reference conditions been defined, where relevant?	1	☑	☑
	Definition of targets and OARs			
14	Has GTV definition been described in sufficient detail, with references if possible?	1	☑	☑
15	Has CTV definition been described in sufficient detail, with references if possible?	1	☑	☑
16	Has the establishment of PTVs (or alternatively robustness settings) been described in sufficient detail?	1	☑	☑
17	Have PTV sizes in the patient cohort been described?	1	☑	☐

18	Have OAR definitions been described in sufficient detail, with references if possible?	1	☑	☑
19	Have PRV margins been described in sufficient detail, with references if available?	1	☑	☑
	Treatment planning system and dose calculation			
20	Have all applied dose calculation algorithms been described in sufficient detail?	1	☑	☑
21	For any commercial software used, have the manufacturer, algorithms and specific versions been stated?	1	☑	☑
22	Have all relevant user parameters and settings in the TPS been reported, e.g. beams, dose grid, control point spacing?	1	☑	☑
23	Have all volumes been evaluated with the same software/methodology?	1	☑	☑
	Planning aims and optimisation			
24	Are clear planning aims defined, including imposed hard constraints and planning objectives (with or without soft constraints)?	5		☑
25	Has the ranking of planning objectives (priorities) been described?	5		☑
26	Is the dose prescription clearly defined?	10		☑
27	Is there a narrative description of the applied optimisation process, including the handling of all objectives with their ranking?	5		☑
28	If manual intervention during or after optimisation is allowed, has this been described?	1	☑	☑
	Bias mitigation			
29	Have enough study details been provided such that bias issues could be noted?	5		☑
30	Has bias been sufficiently mitigated to reliably answer the posed research question?	10		☑
	Plan acceptability – minor and major protocol deviations			
31	Was the procedure for assessment of plan acceptability well-described?	1	☑	☑
32	Was the procedure for assessment of minor and major protocol deviations well described?	1	☑	☑
	Plan (re-)normalisation for plan comparisons			
33	Has plan (re-)normalisation been described sufficiently?	1	☑	☑
	Dose-volume parameters for plan evaluation and comparison			
34	Have sufficiently comprehensive dose-volume parameters been used for plan evaluations and comparisons?	5		☑
	Population-mean DVHs			

35	Has the algorithm for creating population-mean/median DVHs been reported?	1	☑	☑
36	Have the definitions of confidence intervals been included?	1	☑	☑
	Plan evaluations by clinicians			
37	Have clinicians scored plans to assess quality?	1	☐	☐
38	Were plan comparisons by clinicians blinded?	1	☐	☐
	Predicted tumour control probability and normal tissue complication probabilities for plan evaluation and comparison			
39	Have any applied TCP models been described and referenced?	1	☐	☐
40	Have any applied NTCP models been described and referenced?	1	☐	☐
	Plan deliverability and complexity			
41	Have methods used to assess plan deliverability and complexity been described in sufficient detail?	1	☑	☑
	Composite plan quality metrics			
42	Is there a sufficient basis (e.g. in the literature) for any selected composite plan quality metrics?	1	☑	☑
43	Is there an adequate description of the calculation of the composite plan quality metrics?	1	☑	☑
	Planning and delivery times			
44	Has measurement of planning times been described in sufficient detail?	1	☑	☐
45	Has the establishment of delivery times been described in sufficient detail?	1	☑	☐
	Statistical analysis			
46	Have proper statistical methods been used and described in sufficient detail?	5		☑
47	In case of multiple testing for research questions, has this been handled appropriately?	1	☑	☑
	**Questions for Results**			
48	Does the provided data contribute to (at least partly) answering all aspects of the research questions, e.g. plan acceptability, dosimetric quality, deliverability and planning and delivery times?	10		☑
	Dose distribution reporting			
49	Are complete summaries of the dose distributions in the patient cohort provided (low doses, high doses, OARs, PTV, patient, etc.)?	5		☑
50	Are tables and figures optimised to clearly present the results obtained?	1	☑	☑
51	Have the answers to the research questions been illustrated for an example patient by providing dose distributions, DVHs, etc.?	1	☑	☑
	Plan acceptability reporting – minor and major protocol deviations			
52	In case of treatment technique or planning technique comparisons, was plan acceptability reported separately for each technique?	1	☑	☑

53	Has plan acceptability been reported in sufficient detail: how many plans were acceptable, how many were not and for what reasons (e.g. violation of hard constraints, violation of soft constraints, other reasons)?	1	☑	☑
54	Was there adequate reporting of minor and major protocol deviations?	1	☑	☐
	Deliverability and complexity reporting			
55	Has the deliverability of the plans been adequately reported?	1	☑	☐
56	Have plan deliverability and complexity been investigated in sufficient detail in relation to the posed research questions?	1	☑	☐
	Planning and delivery times reporting			
57	Have planning and delivery times been adequately evaluated and reported?	1	☑	☑
	Patient-specific analyses reporting			
58	Is there sufficient description of inter-patient variations in the results presented?	1	☑	☑
59	Have outlier patients been reported and has any exclusion from population analyses been sufficiently motivated and explained?	1	☑	☐
	Statistical reporting			
60	Are the p-values reported appropriately?	1	☑	☑
61	Are there confidence intervals for the appropriate parameters?	1	☑	☑
**Questions for discussions**				
62	Is there an overall interpretation of the data presented in the Results section as to how the posed research questions are answered?	10		☑
	Comparison with literature			
63	Has the study been sufficiently discussed in the context of existing literature?	5		☑
	Clinical and statistical significance			
64	Does the discussion focus on statistically significant results?	1	☑	☑
65	Is the potential clinical significance of the results clearly discussed (assuming practical application would be feasible)?	5		☑
	Clinical applicability of the study			
66	Is future the clinical applicability sufficiently discussed?	1	☑	☑
	Study limitations			
67	Has the impact of the study limitations on the provided answers to the research questions been sufficiently discussed?	10		☑
	Future work			
68	Has the potential future work arising from the study been	1	☑	☑
**Questions for conclusions**				
69	Do the presented conclusions represent answers to the posed research questions?	5		☑

70	Are the conclusions fully supported by the results?	5		☑
71	Are the conclusions a fair summary of all results?	5		☑

Questions for supplementary

Supplementary materials

72	Is the information presented in the supplementary material of sufficient relevance?	1	☑	☑
73	Is the presentation of the included information of sufficient quality, including readability?	1	☑	☑
74	Has sufficient underlying data been made available or a willingness to share data been indicated, within local data sharing restrictions?	5		☑

RATING remarks

75	Is the RATING score added to the manuscript?	5		☑
76	Is the accompanying question table added to the cover letter or the supplementary material?	1	☑	☑

RATING score	96%		
**RATING fraction**	204	of	212

References

[1] Mizowaki T, Takayama K, Nagano K, Miyabe Y, Matsuo Y, Kaneko S, et al. Feasibility evaluation of a new irradiation technique: three-dimensional unicursal irradiation with the Vero4DRT (MHI-TM2000). J Radiat Res. 2013;54(2):330-6. https://doi.org/10.1093/jrr/rrs076.

[2] Burghelea M, Verellen D, Dhont J, Hung C, Gevaert T, Van den Begin R, et al. Treating patients with Dynamic Wave Arc: First clinical experience. Radiother Oncol. 2017;122:347-51. https://doi.org/10.1016/j.radonc.2017.01.006.

[3] Burghelea M, Verellen D, Poels K, Hung C, Nakamura M, Dhont J, et al. Initial characterization, dosimetric benchmark and performance validation of Dynamic Wave Arc. Radiat Oncol. 2016;11:63. https://doi.org/10.1186/s13014-016-0633-7.

[4] Uto M, Mizowaki T, Ogura K, Miyabe Y, Nakamura M, Mukumoto N, et al. Volumetric modulated Dynamic WaveArc therapy reduces the dose to the hippocampus in patients with pituitary adenomas and craniopharyngiomas. Pract Radiat Oncol. 2017;7:382-7. https://doi.org/10.1016/j.prro.2017.04.004.

[5] Ono Y, Yoshimura M, Hirata K, Ono T, Hirashima H, Mukumoto N, et al. Dosimetric advantages afforded by a new irradiation technique, Dynamic WaveArc, used for

accelerated partial breast irradiation. Phys Med. 2018;48:103-10. https://doi.org/10.1016/j.ejmp.2018.03.015.

[6] Hiraoka M, Mizowaki T, Matsuo Y, Nakamura M, Verellen D. The gimbaled-head radiotherapy system: Rise and downfall of a dedicated system for dynamic tumor tracking with real-time monitoring and dynamic WaveArc. Radiother Oncol. 2020;153:311-318. https://doi.org/10.1016/j.radonc.2020.07.002.

[7] Stronger and more flexible X-ray radiotherapy. Hitachi Ltd. Nature Portfolio. 2024 Mar 20. https://www.nature.com/articles/d42473-023-00445-6.

[8] Goto Y, Nakamura A, Ashida R, Sakanaka K, Itasaka S, Shibuya K, et al. Clinical evaluation of intensity-modulated radiotherapy for locally advanced pancreatic cancer. Radiat Oncol. 2018;13:118. https://doi.org/10.1186/s13014-018-1063-5.

[9] Miften M, Olch A, Mihailidis D, et al. Tolerance limits and methodologies for IMRT measurement-based verification QA: Recommendations of AAPM Task Group No. 218. Med Phys. 2018;45(4):e53-e83. https://doi.org/10.1002/mp.12810.

[10] Hansen CR, Crijns W, Hussein M, Rossi L, Gallego P, Verbakel W, et al. Radiotherapy Treatment plannINg study Guidelines (RATING): A framework for setting up and reporting on scientific treatment planning studies. Radiother Oncol 2020;153:67-78.

https://doi.org/10.1016/j.radonc.2020.09.033.
